# Mixed germ cell tumor on an ectopic ovary with in a 9-year-old girl: A rare and complex case

**DOI:** 10.1016/j.radcr.2025.07.047

**Published:** 2025-08-18

**Authors:** Chaimae ABOURAK, Siham Oukassem, Wadie Zouita, Imade Bougrine, Jaouad Bouljrouf, Lina Belkouchi, Nazik Allali, Mounir Kisra, Latifa Chat, Siham El Haddad

**Affiliations:** a Department of Radiology, Mother-Child, Faculty of Medicine and Pharmacy of Rabat, Children's Hospital, Ibn Sina University Hospital, Mohammed V University, Rabat, Morocco; b Department of Surgery, Faculty of Medicine and Pharmacy of Rabat, Children's Hospital, Ibn Sina University Hospital, Mohammed V University, Rabat, Morocco

**Keywords:** Mixed germ cell tumor, Ectopic ovary, Adnexal torsion, Family history of cancers

## Abstract

Malignant ovarian germ cell tumors are rare in the pediatric population, particularly in children under ten years of age. We report a unique case of a mixed germ cell tumor, comprising dysgerminoma, mature and immature teratomas, and yolk sac tumor, located on an ectopic ovary, which was discovered following adnexal torsion in a 9-year-old girl with a family history of gynecological cancers. Mixed germ cell tumors present significant diagnostic challenges due to their heterogeneous histological composition and complex biological behavior. The patient's family history of gynecological cancers raises the possibility of an underlying genetic predisposition. This case underscores the importance of a comprehensive approach to pediatric pelvic masses, particularly in patients with a significant family history of cancer. Long-term follow-up is crucial for the early detection of recurrence in complex germ cell tumors.

## Introduction

Malignant ovarian germ cell tumors are rare, with an incidence of approximately 0.5 per 100,000 individuals [1]. Although these tumors can occur across all age groups, they are most commonly diagnosed in children and adolescents, exhibiting diverse biological behaviors and prognoses [1]. Among them, mixed germ cell tumors are particularly intriguing due to their heterogeneous histological composition, combining various germ cell tumor subtypes, each with distinct clinical and prognostic implications. This case presents a mixed germ cell tumor located on an ectopic ovary—an unusual site—complicated by torsion, in a 9-year-old pediatric patient. The unique clinical presentation is further complicated by a family history of gynecological cancers, raising concerns about potential genetic susceptibility, a factor rarely documented in the literature for this tumor type. This study examines the diagnostic and therapeutic aspects of this complex case, emphasizing the pivotal role of multimodal imaging and tumor markers in the management of atypical pediatric pelvic masses, as well as the essential contribution of histopathological analysis.

## Case presentation

We report the case of a 9-year-old girl, the youngest of 3 siblings, with no significant personal medical or surgical history. She was born from a second-degree consanguineous marriage. Her 2 older sisters are in good health. However, her family history was notable for malignancies on the maternal side: her mother passed away 1 year prior due to metastatic breast cancer, and a maternal cousin died from uterine cancer at a young age.

The patient had experienced intermittent pelvic pain for approximately 1 year. Initially, this was attributed to colopathy by her general practitioner. Over the months, the pain persisted with fluctuating intensity, occasionally interfering with daily activities. Two weeks prior to admission, the symptoms acutely worsened, presenting as severe lower abdominal pain accompanied by intermittent, non-bilious vomiting. There were no associated urinary or bowel disturbances. The severity of symptoms prompted an emergency pediatric consultation and subsequent referral to the pediatric surgical unit at our university hospital.

At admission, the patient was alert and hemodynamically stable. Her vital signs were within normal ranges: heart rate at 82 bpm (normal: 70-110 bpm), blood pressure at 120/80 mmHg, respiratory rate at 16 breaths per minute (normal: 12-20 bpm), and oxygen saturation at 98% on room air. On physical examination, there was tenderness in the lower abdomen with a palpable midline pelvic mass measuring approximately 8 × 6 cm. Bilateral inguinal lymphadenopathy was noted, while the remainder of the systemic examination was unremarkable. There were no signs of bowel obstruction or peritoneal irritation.

Initial laboratory workup showed a normal complete blood count and inflammatory markers. Tumor marker assessment revealed elevated levels:

• **Alpha-fetoprotein (AFP):** 274 ng/mL (normal <10 ng/mL)
• **CA-125:** 333 U/mL (normal <35 U/mL)
• **β-Human chorionic gonadotropin (β-HCG):** 3.1 mIU/mL (normal <5 mIU/mL)
• **Carcinoembryonic antigen (CEA):** 1.97 ng/mL (normal <5 ng/mL) Viral serologies including HIV, hepatitis B, and C were negative.

**Fig. 1:**
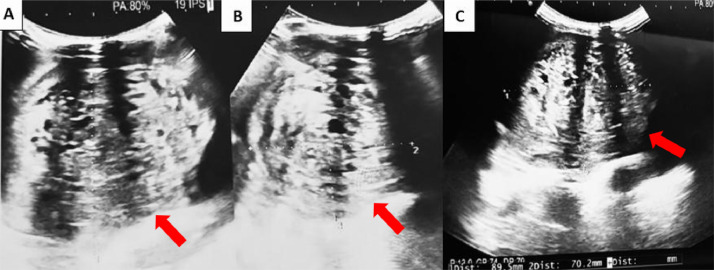
Abdominopelvic ultrasound: Longitudinal (A) and Transverse (B and C) Views. A well-defined, suspicious oval-shaped pelvic mass with regular borders, heterogeneous and hyperechoic, containing several cystic locules (red arrow), measuring 89.5×70.2×66 mm (height x transverse diameter x anteroposterior diameter).

Pelvic ultrasound revealed a well-circumscribed, oval, heterogeneous hyperechoic mass in the midline pelvis, containing cystic areas. The lesion was suspicious for a teratoma or possibly a rhabdomyosarcoma (Fig. 1).

**Fig. 2:**
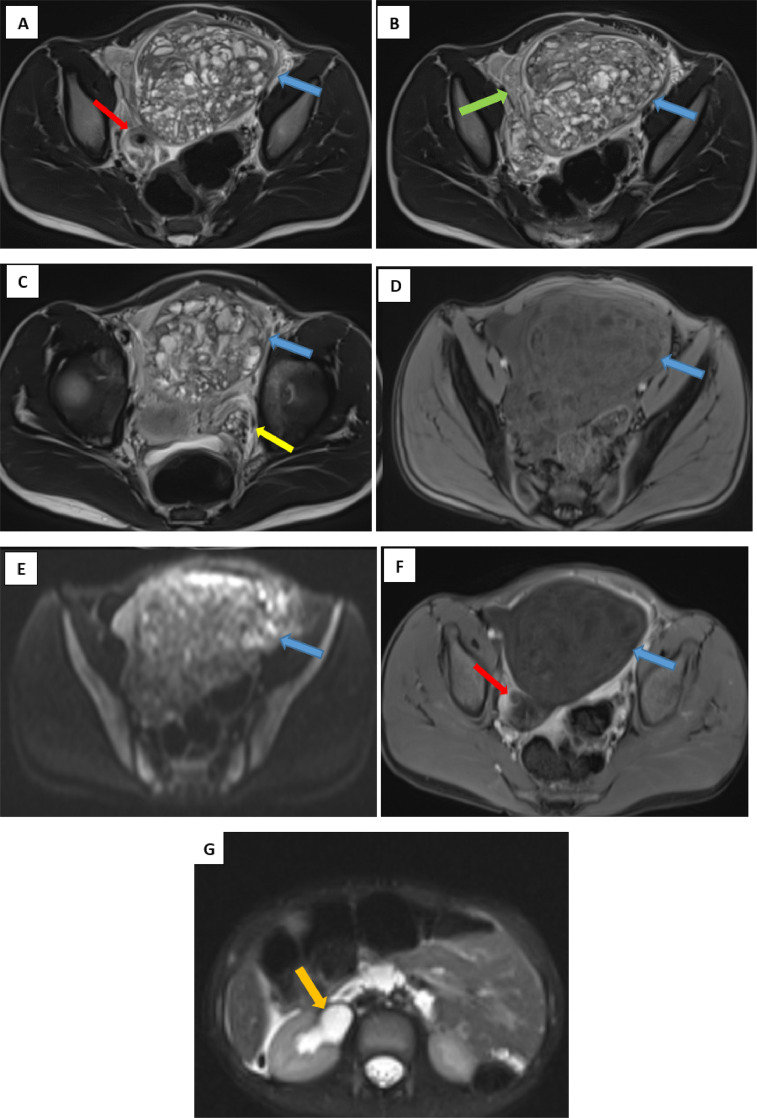
Abdominopelvic MRI. Axial views: T2-weighted sequence (A, B, C), T1-weighted sequence (D), diffusion-weighted imaging (E), contrast-enhanced T1-weighted sequence with fat suppression (F), and T2-weighted fat-saturated HASTE sequence (G). A large pelvic mass (blue arrow) is visualized, showing the "spurring" sign with the right ovary, which exhibits peripheral follicular distribution and stromal edema (green arrow). The mass is well-circumscribed, lobulated, and heterogeneous, with both cystic and solid components. It appears isointense on both T1- and T2-weighted images. Diffusion-weighted imaging reveals restricted diffusion, and there is an absence of enhancement of both the mass and the residual ovarian parenchyma following gadolinium injection. A twisted pedicle and ipsilateral fallopian tube are also noted (red arrow), along with upstream hydroureteronephrosis (orange arrow). The left ovary (yellow arrow) is of normal size and morphology, with multiple follicles.

Given the chronicity of symptoms, the stability of the patient’s condition, and the suspicious imaging findings, pelvic MRI was performed for further characterization. MRI showed a large right pelvic mass with lobulated margins, demonstrating mixed solid and cystic components. It was isointense on both T1- and T2-weighted images with areas of restricted diffusion and no post-contrast enhancement. A "beak sign" indicated continuity with the right ovary, which was enlarged and exhibited peripheral follicles and stromal edema. The presence of a twisted vascular pedicle and fallopian tube—the “whirl sign”—suggested adnexal torsion (Fig. 2A–F). The torsion was also responsible for ipsilateral ureterohydronephrosis due to ureteral compression (Fig. 2G).

Given the imaging findings and the elevated tumor markers, the case was discussed in a multidisciplinary tumor board. Surgery was indicated due to the presence of a non-metastatic secreting ovarian mass, with a decreasing trend in AFP observed on early monitoring (dropping to 105.6 ng/mL).

**Fig. 3:**
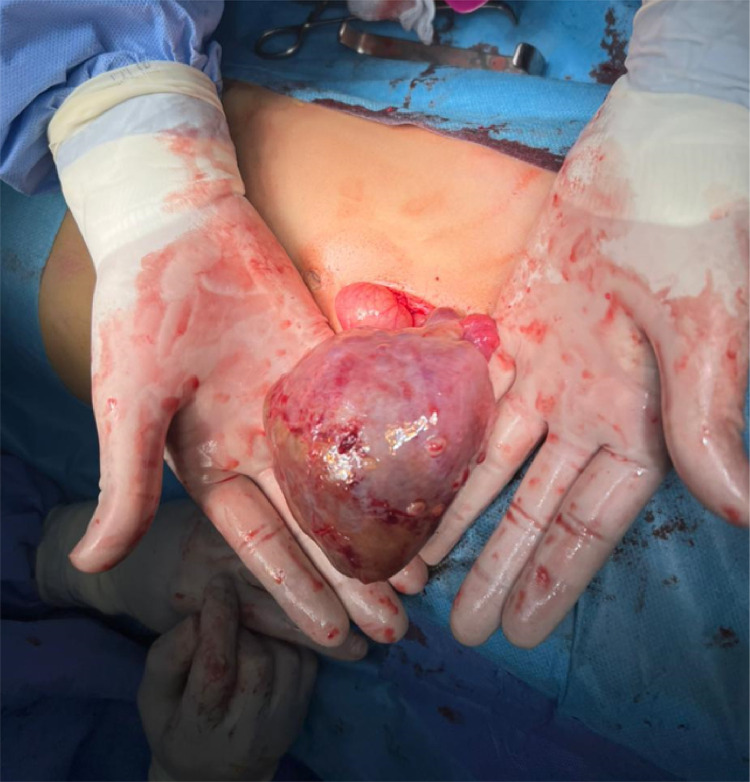
Intraoperative photograph. The excised tumor appears encapsulated, ovoid in shape, and well-demarcated. Its external surface is smooth and shiny, with a whitish to grayish coloration. Superficial vascularization is visible, with no macroscopic evidence of infiltration into adjacent tissues.

During exploratory laparotomy, surgeons discovered a large mass attached to the right fallopian tube. The right ovary was not visualized at its expected anatomical location, and sero-hematic fluid was found in the pelvis (Fig. 3). The mass was excised and sent for pathological examination.

Macroscopically, the mass was grayish-white, partially hemorrhagic and multicystic, with yellowish and whitish areas. Microscopically, it showed a mixed germ cell tumor with multiple components:

• **Mature teratoma:** including cartilage, fibroadipose tissue, and keratinizing squamous epithelium.
• **Immature teratoma component:** estimated at 10%.
• **Dysgerminoma component:** estimated at 30%, immunohistochemically positive for placental alkaline phosphatase (PLAP).
• **Yolk sac tumor component:** with microcystic architecture, estimated at 10%.

**Fig. 4:**
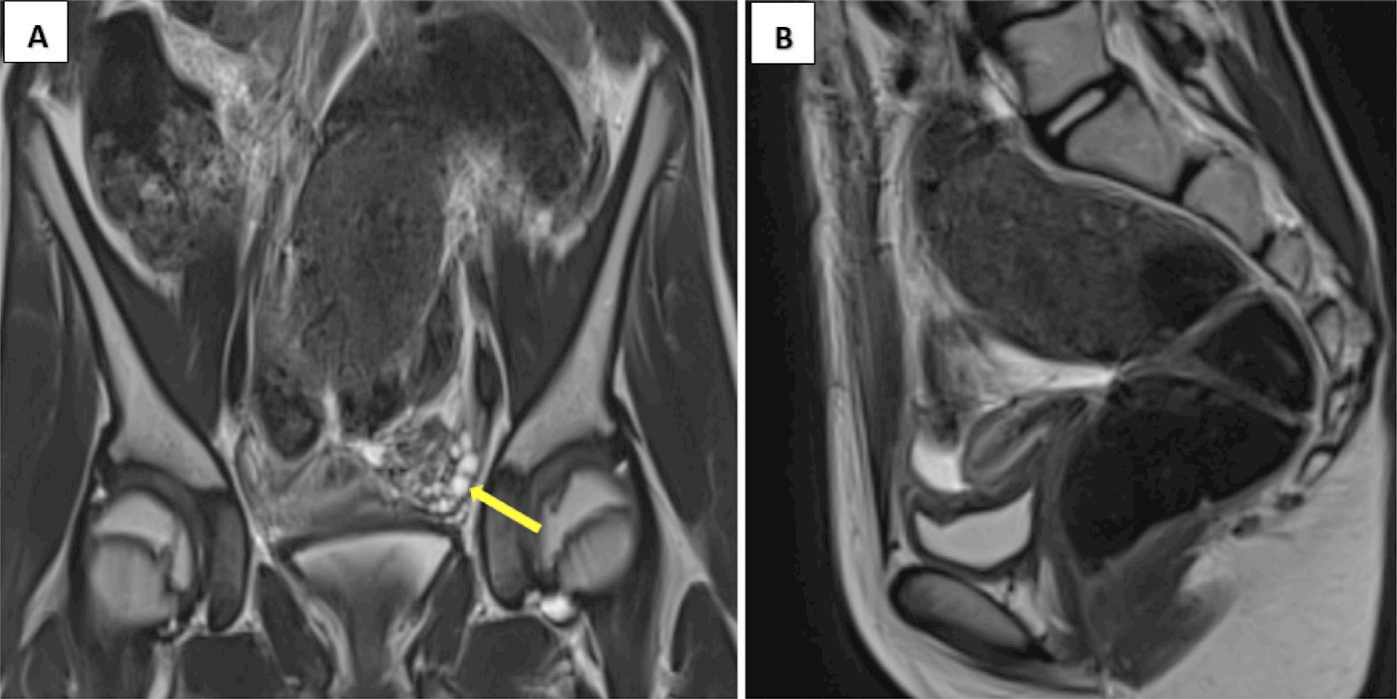
Postoperative abdominopelvic MRI. Coronal (A) and sagittal (B) views in T2-weighted sequences. The surgical bed appears clear, with no evidence of residual mass. The left ovary (yellow arrow) is of normal size and morphology, with a follicular pattern.

The pathological diagnosis confirmed a **mixed malignant germ cell tumor arising** from an ectopic ovary. Postoperative AFP decreased further to 12.81 ng/mL. A follow-up pelvic MRI (Figs. 4A and B) demonstrated complete resection with no residual mass. The patient was placed under close oncologic and gynecologic follow-up. Given the significant family history of gynecological malignancies, genetic counseling and testing were recommended.

At 3- and 6-month follow-up visits, the patient remained asymptomatic with no radiologic or biochemical evidence of recurrence.

## Discussion

Ovarian tumors are classified into epithelial neoplasms, mesenchymal neoplasms, sex cord-stromal tumors, and germ cell tumors [2]. Germ cell tumors account for 15%-20% of all ovarian neoplasms [3]. They originate from the primordial germ cells of the embryonic gonad [3,4]. According to the World Health Organization’s 2020 classification, ovarian germ cell tumors include mature and immature teratomas, dysgerminoma, yolk sac tumors, embryonal carcinoma, non-gestational choriocarcinoma, mixed germ cell tumors, monodermal and somatic-type teratomas, and sex cord-stromal germ cell tumors [2,3]. All ovarian germ cell tumors are malignant, except for mature teratomas [3].

Malignant ovarian germ cell tumors constitute less than 5% of all ovarian neoplasms [[3], [4], [5]]. Although rare overall, malignant mixed germ cell tumors are the most common type of malignant ovarian neoplasm in young girls and adolescents, particularly in the second decade of life [[3], [4], [5]]. Our patient, being 9 years old, makes this case rare. These tumors are fast-growing and may metastasize [3]. Fortunately, in our patient, the tumor was large but non-metastatic. In children and adolescents, more than 60% of ovarian neoplasms are of germ cell origin, with about one-third being malignant [3]. In adults, the vast majority of germ cell tumors are benign (mature cystic teratomas) [3].

Malignant mixed germ cell tumors consist of at least 2 germ cell neoplasms, with at least one being a malignant germ cell tumor, and represent 5 to 20% of all malignant germ cell tumors [3,4,6,7]. These tumors are characterized by the presence of 2 or more malignant germ cell components [[3], [4], [5],^7^]. Ovarian mixed germ cell tumors typically contain 2 or more malignant germinal components. The most common component in mixed malignant germ cell tumors (MMGCT) is dysgerminoma (80%), followed by yolk sac tumor (70%), immature teratoma (53%), choriocarcinoma (20%), and embryonal carcinoma (16%). [6] The combination of dysgerminoma and yolk sac tumor is the most frequent mixture. [3,7]

The presence of multiple histological subtypes, as seen in our case (with components of dysgerminoma, teratoma, and yolk sac tumor), is relatively rare and confers a more complex prognosis due to the varied behaviors of each component [4,6]. Dysgerminomas, for example, have relatively low malignancy on their own but can be associated with more aggressive components, necessitating a rapid multidisciplinary approach to prevent progression [4,8].

The initial presentation of intermittent pelvic pain, initially interpreted as irritable bowel syndrome, is common and may delay diagnosis, as observed in our patient. Progression to acute pain with vomiting, ultimately leading to the diagnosis of adnexal torsion, is also characteristic of complex ovarian tumors. The presence of a mass can cause ovarian pedicle rotation, resulting in ischemic complications and, in some cases, hydronephrosis due to ureteral compression, as seen in our patient [2,4].

In our case, ultrasound and MRI identified a mass with both cystic and solid components, typical of a germ cell ovarian tumor. MRI signal characteristics, combined with an elevated AFP level, strongly suggested a germ cell origin, consistent with findings in other cases of malignant mixed tumors in young patients [2,3,6].

Tumor markers are essential for diagnosing and monitoring ovarian germ cell tumors. An elevated AFP level, as observed in our patient, is typically associated with yolk sac tumors, while CA 125 is more commonly elevated in epithelial tumors, though it may also appear in certain complex germ cell tumors [1]. The post-resection decline in AFP levels is a reliable indicator of treatment response, as demonstrated in our case.

Surgical management of malignant ovarian germ cell tumors in children typically involves tumor resection or adnexectomy, with fertility preservation being prioritized whenever possible, in accordance with current recommendations [1]. In this case, laparotomy allowed for complete resection, and histopathological examination revealed multiple tumor components, providing a definitive diagnosis.

The family history of gynecological cancers, including breast and uterine cancer, raises concerns about potential genetic susceptibility. Although rare, ovarian germ cell tumors have been reported in carriers of BRCA1/2 mutations [8]. A genetic study was requested for this patient to evaluate any possible hereditary risk. Identifying BRCA mutations or other susceptibility genes could influence long-term follow-up and guide preventive measures for family members.

Ovarian germ cell tumors in children generally have a favorable prognosis, with high survival rates, particularly when the tumor is localized and metastasis-free at the time of diagnosis. Close monitoring through MRI and AFP levels is recommended to detect any recurrence, especially for mixed tumors, which have a higher recurrence potential than purely benign germ cell tumors [1,9].

Recent studies underscore the importance of prognostic factors such as tumor size, serum marker levels, and histological composition in the management of these tumors [4,7]. While the absence of recurrence at 3- and 6-month follow-up is reassuring, long-term monitoring is recommended due to the tumor's histological complexity.

## Conclusion

This case highlights a rare mixed germ cell tumor on an ectopic ovary complicated by torsion. The clinical and paraclinical findings emphasize the importance of early diagnosis and a multidisciplinary approach for young patients presenting with persistent pelvic pain. Furthermore, rigorous long-term follow-up is essential to monitor for recurrence and ensure optimal management.

## Patient consent

Written informed consent for the publication of this case report was obtained from the parent and guardian.

## References

[1] Solheim O., Nilsen S.M., Abeler V.M., Tropé C.G., Kristensen GB. (2014). Prognostic factors in malignant ovarian germ cell tumours (The Surveillance, Epidemiology and End Results experience 1978–2010). Eur J Cancer.

[2] Amante S., Félix A., Cunha TM. (2023). Ovarian dysgerminoma: clues to the radiological diagnosis. Diagn Interv Radiol.

[3] Rana S., Sood N., Garg B., Singh S. (2016). Immature teratoma with embryonal carcinoma: a rare malignant mixed germ cell tumor in a 13-year-old girl. Iran J Pathol.

[4] Goyal L.D., Garg S., Suri V., Goyal A., Rajwanshi A., Gupta N. (2014). Malignant mixed germ cell tumour of the ovary: an unusual combination and review of literature. J Ovarian Res.

[5] Bel Haj Salah M., Bdioui A., Khalfallah M., Hmissa S., Mokni M. (2010). Tumeur germinale mixte de l’ovaire avec composante rhabdomyosarcomateuse: à propos d’un cas. Ann Pathol.

[6] Patel T., Gandhi S., Kantharia V., Desai S. (2022). A rare case of ovarian gonadoblastoma flourishing into malignant mixed germ cell tumor with review of literature. Int Cancer Conf J.

[7] Kwon M.J., Roh J., Kim HS. (2009). Bowel loop in an ovarian tumor: grossly visible, completely developed intestinal loop in mature cystic teratoma of malignant mixed germ cell tumor. Pathol Int.

[8] Hamel N., Tonin P.N., Provencher D., Mes-Masson A.M., Ghadirian P., Foulkes WD. (2007). Ovarian germ cell tumor in a BRCA2 mutation carrier. Int J Gynecol Pathol.

[9] Tsutsumi Y., Yamashita M., Kurihara K., Yamaguchi Y., Arita K. (2020). An aggressive systemic mastocytosis preceded by ovarian dysgerminoma. BMC Cancer.

